# Α Multicenter Retrospective Study Evaluating Brivaracetam in the Treatment of Epilepsies in Clinical Practice

**DOI:** 10.3390/ph14020165

**Published:** 2021-02-19

**Authors:** Maria Stefanatou, Eirini Vasileiadou Kapetanou, Vasilios K. Kimiskidis, Vasileios Papaliagkas, Panagiotis Polychronopoulos, Sofia Markoula, Kleoniki Charisiou, Dimitrios Kazis, Anastasia Verentzioti, Panayiotis Patrikelis, Athanasia Alexoudi, Stylianos Gatzonis

**Affiliations:** 1 1st Department of Neurology, National and Kapodistrian University of Athens, 115 28 Athens, Greece; 2 Department of Applied Statistics, University of Piraeus, 185 34 Piraeus, Greece; eirinivk@gmail.com; 3 Laboratory of Clinical Neurophysiology, Aristotle University of Thessaloniki, 541 24 Thessaloniki, Greece; kimiskid@auth.gr (V.K.K.); vpapal@auth.gr (V.P.); 4 Department of Neurology, University of Patras, 254 04 Patras, Greece; polychpan@upatras.gr; 5 Department of Neurology, University Hospital of Ioannina, 451 10 Ioannina, Greece; smarkoula@uoi.gr (S.M.); xarisiouklairie@yahoo.com (K.C.); 6 3rd Department of Neurology, “G. Papanikolaou” Hospital, Aristotle University of Thessaloniki, 541 24 Thessaloniki, Greece; dimitrios.kazis@gmail.com; 7 1st Department of Neurosurgery, “Evangelismos” Hospital, National and Kapodistrian University of Athens, 106 76 Athens, Greece; nverentzioti@gmail.com (A.V.); ppatrikelis@gmail.com (P.P.); alexoudath@yahoo.gr (A.A.); sgatzon@med.uoa.gr (S.G.)

**Keywords:** epilepsy, treatment, antiepileptic drugs, brivaracetam, clinical practice

## Abstract

Brivaracetam (BRV) is the latest approved antiepileptic drug. The aim of the study was to evaluate the efficacy and tolerability of BRV in everyday clinical practice. In this retrospective, observational, multicenter study, data from epilepsy patients receiving BRV from January 2018 to July 2019 were analyzed. Patients with age ≥16 suffering from any type of epilepsy and having at least one follow up encounter after dose titration were included. 156 consecutive patients were included in the study. The mean age was 40 (16–84 years) and the mean duration of epilepsy was 21 years. Of the 156 patients, 81% were diagnosed with focal-onset seizures, 16% with generalized seizures, while 3% suffered from unclassified seizures. Nine patients received BRV as monotherapy as a switching therapy. At the first follow up visit, seizure cessation was achieved in 56 (36%) patients and the rate of ≥50% responders was 36%. Twenty four patients (15%) remained unchanged; six patients (4%) were recorded with increased seizure frequency, while the remaining 9% had a response of less than 50%**.** Twenty-six patients (17%) showed clinically significant adverse events, but none were life threatening. Brivaracetam seems to be an effective, easy to use and safe antiepileptic drug in the clinical setting.

## 1. Introduction

Epilepsy is one of the most common neurologic diseases, affecting over 65 million people worldwide [1]. Despite the expansion of the antiepileptic-drug (AED) repertoire, almost one-third of epilepsy patients are reluctant to continue treatment long term [2]. Furthermore, many patients suffer from side effects of AEDs, which are responsible for the poor adherence and discontinuation of therapy, resulting in increased risk of uncontrolled seizures and death [3]. Therefore, research aimed at developing new AEDs with improved efficacy and tolerability profiles continues.

Brivaracetam (BRV) is the latest approved antiepileptic drug and acts as a high-affinity synaptic vesicle protein 2A (SV2A) ligand that exceeds the binding potential of levetiracetam (LEV) [4]. In the European Union, BRV is only approved as an adjunctive therapy for the treatment of partial-onset seizures (POS) with or without secondary generalization, in patients 16 years of age or older, with epilepsy [5]. In Phase III clinical trials, BRV (50–200 mg/day) has shown efficacy and an adequate safety profile in patients suffering from POS [6,7,8,9]. In additional open-label studies, BRV has demonstrated promising results and high retention rates in patients with both focal-onset and genetic generalized epilepsies [10,11,12]. The main treatment-emergent adverse events (TEAEs) observed during the regulatory trials were somnolence, dizziness and headache, followed by fatigue and nausea [12].

However, results from clinical trials may not be always reproducible in everyday practice, as these studies have limitations arising from their short duration and strict inclusion and exclusion criteria excluding some subgroups of epilepsy patients. They also adhere to strict dosing protocols, allowing little, if any, dosing flexibility [13,14,15]. Given the above, there are concerns about the potential efficacy and tolerability of a new drug, such as BRV, in daily clinical practice.

The present post-marketing study was designed to collect data on the effectiveness and safety of brivaracetam in patients with epilepsy in everyday clinical practice.

## 2. Results

In total, 156 consecutive patients (82 males and 74 females) were included in the study. The mean age of patients was 40 years (five patients between 16 and 18 years-old) and the mean duration of the disease was 21 years. Nine patients (6%) received BRV as monotherapy.

### 2.1. Demographic and Clinical Data

The demographic and clinical characteristics of the participants are shown in Table 1.

### 2.2. Brivaracetam and Levetiracetam

As shown in Table 2, 102 patients (65%) were treated with levetiracetam (LEV) in the past and 85 of them at the baseline visit. The reasons for LEV withdrawal included unresponsiveness (38, 5%), adverse events (17%) or both (10%).

### 2.3. Responsiveness

The rate of ≥50% responders after BRV initiation was 71% (112 patients), 36% (56 patients) of them reported no seizure activity; while 15% (24 patients) remained unchanged and 4% (six patients) demonstrated an increase in seizures (Table 3).

**Figure 1:**
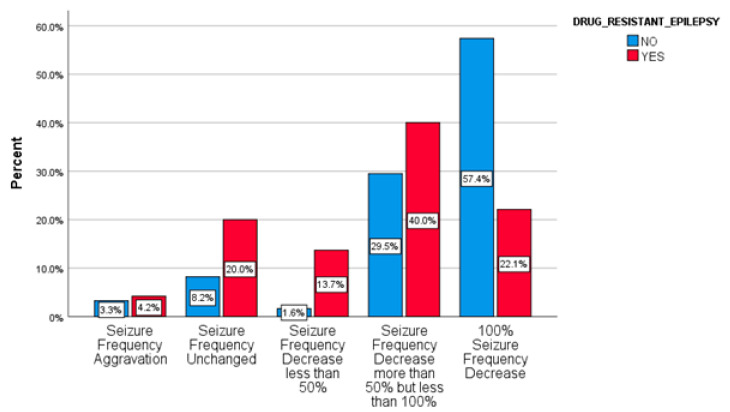
Response rates in drug-resistant epilepsy.

The ≥50% response rate after BRV initiation treatment in the subgroup of patients with drug-resistant epilepsy was 62% (22% of them achieved seizure freedom) (Figure 1). A different response rate was recorded between the two subgroups, but this was not statistically significant (chi^2^ = 2.973, *p* = 0.085).

**Figure 2:**
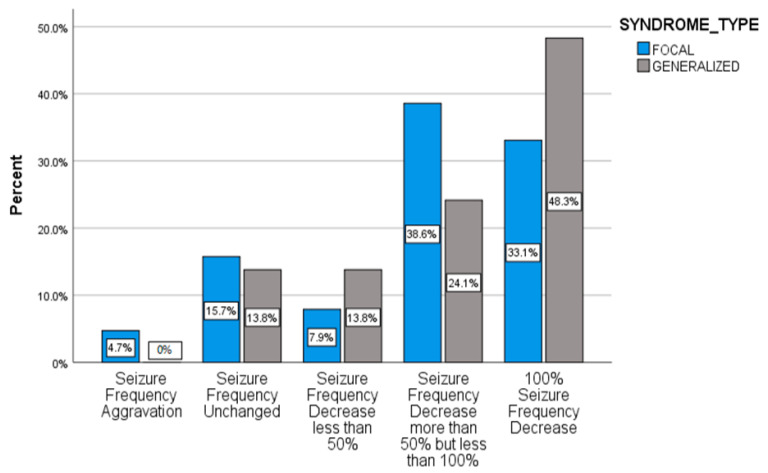
Response rates after BRV initiation correlated to epilepsy syndrome.

No significant difference in the effectiveness of BRV treatment in regard to focal or generalized epilepsy syndrome was recorded (*p* = 0.812) (Figure 2).

Specifically, the ≥50% BRV response in patients switched from LEV was 79% (30% seizure free), as shown in Table 4.

**Figure 3:**
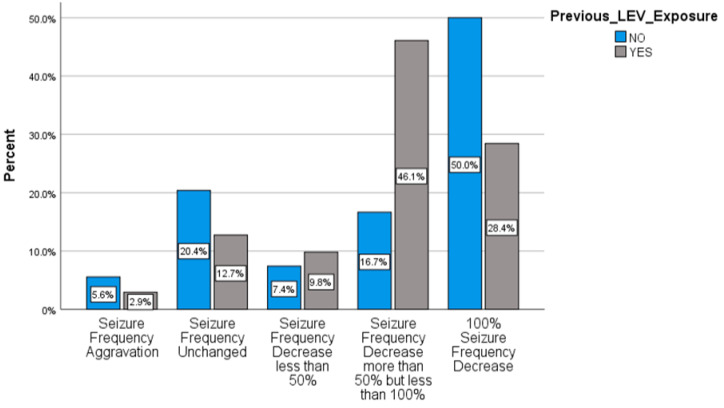
Response rates after BRV treatment in LEV + and LEV− subgroups.

Comparisons of responsiveness measures in the previous LEV exposed (LEV+) and LEV naive (LEV–) subpopulations demonstrated significant differences in outcome categories (chi^2^ = 13.596, *p* = 0.0001) (Figure 3).

As seen in Table 2, the mean dose of BRV intake was 172 mg (median: 200 mg) and the dose range was 50–300 mg.

**Figure 4:**
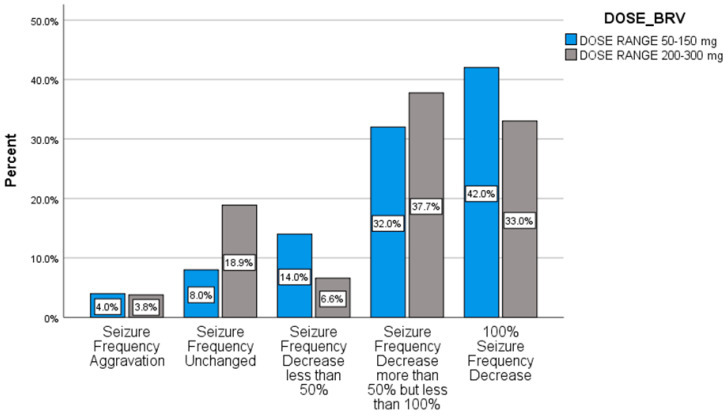
Response rates after BRV treatment correlated to BRV dose.

Comparing the responsiveness of BRV based on dose, we recorded no significant effect in seizure frequency between the two groups (ch^2^ = 2.477, *p* = 0.111) (Figure 4).

Age, gender, previous AED and reason for BRV indication had no influence on the BRV response.

### 2.4. Treatment-Emerged Adverse Events

Twenty-six patients (17%) showed clinically significant adverse events, none of which were life threatening. Among them, 18 patients had non-behavioral side effects (headache, fatigue, etc.) and eight patients suffered from behavioral changes (such as aggressiveness and depression). The most frequent events were somnolence (six patients—4%) and fatigue (two patients—1, 4%). Sixteen patients discontinued BRV after the first follow up. The reasons for discontinuation were lack of efficacy (two patients), adverse events (10 patients) or both (four patients) (Table 5).

In the group switching from LEV, 10 patients (12%) suffered from adverse events with three (5%) of them displaying behavioral adverse events (BAEs—two of them were suffering from irritation and one from anxiety) (Table 6). The most common adverse events (AE) in this group were dizziness (3, 5%) and somnolence (3, 5%), while one patient presented diarrhea and two suffered from headache.

## 3. Discussion

Our multicenter study reflects the real-life experience of on and off-label BRV use in a cohort of 156 patients with different epilepsy syndromes, variations in seizure burden, different concomitant antiepileptic drugs and comorbidities. The study took place in the outpatient epilepsy clinics of five tertiary teaching hospitals, where the patients were followed up by expert epileptologists.

Sixty five percent of the cohort had already received treatment with LEV and 83% of those (85/102) were on LEV treatment at their baseline visit.

Our efficacy rates, with a ≥50% responder rate of 71% (36% seizure-free) at the first follow up visit, are higher than those observed during previous randomized, double-blind controlled trials (RCTs) [6,7,8,9]. Three of these phase III studies (N01252^6^ 20/50/100 mg/day, and N01253^7^ 5/20/50 mg/day; N01358^8^ 100/200 mg/day) applied fixed-dose regimens and only included patients with focal epilepsies, whereas one study (N01254^9^) was designed for treatment with individual doses (20–150 mg/day) and comprised patients with focal (90%) and generalized (10%) epilepsies. These RCTs showed 50% responder rates between 32.7 and 55.8% for 50 mg/day, 36–38.9% for 100 mg/day, and 37.8% for 200 mg/day, respectively.

This result was surprising since most of the patients were receiving or had received LEV treatment in the past although, as expected, the difference in response rates in LEV+ and LEV− subpopulations was statistically significant. This could be explained by the similar mechanism of action. It is well known from previous studies [9,13], that LEV exposure has been associated with lower BRV response rates. However, more recent data provide results similar to our rates, with higher ≥50% response rates in patients already receiving treatment with LEV [12,16,17,18]. It is notable that 26 patients (30%) from the switching group remained seizure free, which was an unexpected result.

The two drugs share a common mechanism of antiepileptic action, but BRV is a high-affinity synaptic vesicle protein 2A (SV2A) ligand that exceeds the binding potential of LEV by 10- to 30-fold [19], which could explain the high response rates, even in the patients with previous LEV exposure.

In a recent study with a longer follow up period (12 months/f/u), the response rates were at the lower end of the effectiveness range of BRV [20]. This may be partially explained by the design of the study, which categorized responders when clinical records only contained qualitative descriptions of seizure improvements rather than a specific quantitative measure of seizure reduction: in that study, such cases were included in the ≤50% improvement group. It is also notable that in some subgroups, the dose of BRV was very low (range 20–50 mg), which could affect the final outcome. In any case, the lower final response rate could reflect the developing tolerance of the drug over time.

In our study, there was no specific classification based on the intellectual ability of the patients. Nevertheless, recent data on the tolerability and efficacy of BRV in patients with intellectual disability and epilepsy, are encouraging [21]. Seizure reductions and no side effects were reported in more than half of all patients. Retention rates for BRV were 84.4 and 58.1% after 3 and 12 months, respectively. Seizure reductions and side effects did not differ significantly between the groups with or without previous LEV treatment.

In a recent study on BRV use for generalized epilepsies [12], Fonseca et al. commented on the higher response rates in observational clinical studies compared to the clinical trials. The hypothesis of overdosing was proposed in terms of flexible dosage in every day clinical setting compared to the strict dosage scheme of RCTs. However, as seen in Figure 4, our analysis showed no dose-dependent outcome.

Another finding from our results is the high seizure-free and responder rates in different epilepsy syndromes, which were included in initial trials. These results are in accordance with recent studies [12,20], which show satisfying outcomes across epilepsy syndromes, providing real life evidence. The molecular similarity to LEV has driven interest towards its use in Genetic Generalized Epilepsy (GGE). During preclinical development, BRV demonstrated suppression of spike-and-wave activity in the genetic absence epilepsy rat Strasbourg model [22]. In humans with GGE, an important reduction in photo paroxysmal response was recorded after BRV treatment [23]. One of the phase III clinical trials included 36 patients with GGE, in which higher responder and seizure free rates were reported compared to the placebo [9]. A retrospective study from Germany also recorded a responder rate of 52.6 and 15.8% of patients who remained seizure-free at 3 months [16]. The different design, the refractory nature of the patients’ profiles and the lack of electroencephalography (EEG) data represent some of the major limitations of the published results. However, the preliminary data suggest that BRV might be an option for GGE.

In our study, BRV was relatively well tolerated with a low rate of adverse events (17%). In line with RTCs and previous data [6,7,8,9,10,11,12,13], the most common TAEs were somnolence, dizziness and behavioral adverse events (such as irritation, aggressiveness and depression). The lower rate of AEs in our study can be explained by the short duration of the follow up period. The consecutive effect of the drug and the increased rates of adverse events over time is well recognized [8]. However, treatment-emergent side effects were the main reason for BRV discontinuation (14 patients out of a total of 16 patients).

In addition to published data regarding BRV tolerance [11,12,24], in our study, the small proportion of behavioral changes in the switching group from LEV suggests that BRV may be an option for patients who have had discontinued LEV due to BAEs. It is known that BRV differs from LEV in that it does not show modulatory activity at α-amino-3-hydroxy-5-methyl-4-isoxazolepropionic acid (AMPA) receptors [25], whereas LEV does [26], suggesting that BRV has a distinct pharmacological profile compared with LEV. The different effects on AMPA receptors can be a likely explanation for the different psychotropic effects of the two drugs.

The better tolerability of BRV compared to LEV, combined with the same or better responsiveness, has been consistently reported in published clinical series in a variety of epileptic syndromes [12,17,18,19], indicating that BRV is a suitable treatment for different epilepsies.

The first limitation of our study is the retrospective design which could result in under-detection and underestimation of TAEs. In addition, the short duration of the follow up period could lead to a bias, since some adverse events could occur after the first six months of treatment, as previously mentioned. Furthermore, the effectiveness of BRV could be lower following long-term use, as there is evidence of development of drug resistance over time.

## 4. Materials and Methods

### 4.1. Study Design

This is a retrospective, multicenter study that collected information from epilepsy outpatient clinics of five tertiary teaching hospitals as follows: (a) Laboratory of Clinical Neurophysiology, Aristotle University of Thessaloniki, “AHEPA” Hospital (b) Department of Neurology, University Hospital of Patras, (c) Department of Neurology, University Hospital of Ioannina, (d) 3rd Department of Neurology, “G. Papanikolaou” Hospital, Aristotle University of Thessaloniki, (e) 1st Department of Neurosurgery, National and Kapodistrian University of Athens, “Evangelismos” Hospital.

In this observational study, data from epilepsy patients receiving BRV anytime between January 2018 and July 2019 were analyzed. Patients aged ≥16 years suffering from all types of epilepsy and having at least one follow up (1–6 months) following dose titration were included.

Drug resistance was assessed according to the 2010 ILAE Task Force guidelines [27]. All patients had a consultant neurologist-confirmed diagnosis of epilepsy and attended specialist follow-up (FU). There was no minimum treatment duration—all patients who had received at least one dose of BRV were included. Patients with no definite diagnosis, without reliable records (such as seizures in bursts or non-epileptic events) and with lack of follow-up information were excluded from the current investigation.

Data collection, storage and management were in compliance with good clinical practice and in accordance with the Declaration of Helsinki. The protocol was approved by the local Ethical Committee of Evangelismos Hospital, NKUA (No: 218/21/8/2018).

### 4.2. Study Cohort

The treating epileptologist at each study site provided information on epilepsy syndromes, etiology, onset of epilepsy, epilepsy duration, semiology, demographics, family and medical history, comorbidities, concomitant and previous AED (any change in other AEDs while on BRV), with detailed history on the use of LEV, using a standardized reporting form.

Patients were asked at each study visit whether they had experienced any adverse events, and they could also spontaneously report adverse events. Behavioral adverse events, such as agitation, antisocial reaction, anxiety, apathy, depersonalization, depression, emotional lability, euphoria, hostility, nervousness, neurosis, and personality disorders, were recorded in detail, as described by Yates et al. [11].

Subpopulations of interest were analyzed by comparing patient groups of LEV+ versus LEV−, drug resistant and drug responsive groups, groups based on BRV dosage and groups of patients with generalized versus focal epilepsy syndrome.

The follow up visits ranged from one month to one year and were performed by an expert epileptologist.

### 4.3. Treatment Regimen

Brivaracetam was administered at the baseline visit. Using a standardized reporting form we recorded starting, maintenance, and maximum doses of BRV, length of exposure to BRV, and withdrawal rates. In the majority of the cases, the target dose was started without a titration phase and the switching from LEV took place directly. In the switching group, the dose target corresponded to previous LEV dosage. In naïve LEV patients, target doses were tailored to individual patient’s needs as assessed by the specialist.

The responsiveness and tolerability of BRV were recorded in detail using seizure diaries and medical records. Efficacy was assessed using responsiveness parameters (≥50% response, <50% response) and seizure frequency.

To ensure standardization, accuracy, and completeness, each patient’s data were reviewed by the study coordinator.

### 4.4. Data Analysis

Data were analyzed using IBM SPSS Statistic, version 25.0 (IBM Corp., Armonk, NY, USA). For subgroup analyses, baseline characteristics were compared using the Pearson chi-square test for qualitative variables. There was no sample size calculation and all analyses were descriptive. Efficacy and adverse events were calculated using data available from the first and the last follow up. We performed linear trend tests to examine the statistical difference in the response rate for LEV+ and LEV− subgroups, drug-resistant and drug-responsive patients, focal and generalized epilepsy syndromes and changes in seizure frequency correlated to BRV dose. *p*-Values of <0.05 were considered as significant.

## 5. Conclusions

Seizure control can be achieved under BRV treatment in different epileptic syndromes. BRV has surprising high response rates with an important proportion of patients remaining seizure free, although the follow up period examined here was short. It also seems to have a good safety profile, especially in patients with previous intolerance to LEV. Therefore, BRV seems to be an effective, easy to use and safe antiepileptic drug to be used in everyday clinical practice.

## Figures and Tables

**Table 1:** Patients’ Characteristics. (M: median, SD: standard deviation).

Characteristics	Baseline, n = 156 (%)
**Age (years), M(SD)**	39.69 (13.63)
**Gender**	**n = 156 (%)**
-Male	82 (52.6)
-Female	74 (47.4)
**Period of follow-up (months), M(SD)**	3.9 (2.76)
**Epilepsy Duration (years), M(SD)**	21.43 (13.66)
**Drug Resistant Epilepsy**	**n = 156 (%)**
-Yes	61 (39.1)
-No	95 (60.9)
**Cosponsored AED, M(SD)**	2.28 (1.40)
**BRV Monotherapy**	9 (5.8)
**Types of Seizures**	**n = 156 (%)**
-Focal Seizures	88 (56.41)
-Focal Seizures with secondary generalization	39 (25)
-Generalized Seizures	25 (16.03)
-Unclassified Seizures	4 (2.56)
**Syndromes**	**n = 156 (%)**
-Idiopathic Epilepsy	69 (44.2)
-Symptomatic Epilepsy	71 (45.5)
-Juvenile Myoclonic Epilepsy	4 (2.6)
-Special Syndromes	12 (7.7)

**Table 2:** BRV and levetiracetam (LEV) Characteristics.

BRV in mg	n = 156 (%)
50	6 (3.8)
100	27 (17.3)
150	17 (10.9)
200	105 (67.3)
300	1 (0.6)
**Mean BRV Dose in mg, M(SD)**	172.12 (46.57)
**Median BRV Dose in mg, M(SD)**	200
**Reasons for BRV Prescription**	**n = 156 (%)**
- Seizures	119 (76.3)
- Adverse Events	23 (14.7)
- Both	14 (9)
**LEV medication at BL**	**n = 156 (%)**
**Treated with LEV in the past**	102 (65.38)
**LEV intake at BL**	85 (83.33)
-Immediate switch to BRV	83 (97.64)
-Gradual switch	2 (2.36)
**LEV in (past) medical history**	17 (16.6)
**LEV naive**	54 (34.61)
**Mean LEV dose in mg, M(SD)**	1278.85 (1326.21)
**Median LEV dose in mg, M(SD)**	1000
**Discontinuation reasons of LEV**	**n = 156 (%)**
- Seizures	60 (38.5)
- Adverse Events	27 (17.3)
- Both	15 (9.6)
- No previous treatment	54 (34.6)

**Table 3:** Seizure frequency percentage change after BRV treatment.

Seizure Frequency Change after BRV Treatment	n = 156 (%)
- Seizure Frequency Unchanged	24 (15.4)
- Seizure Freedom	56 (35.9)
- Seizure Frequency Aggravation	6 (3.8)
- Seizure Frequency Responder < 50%	14 (9)
- Seizure Frequency Responder ≥ 50%	56 (35.9)
- Seizure Frequency Unchanged	24 (15.4)

**Table 4:** BRV response after switching from LEV treatment.

Seizure Frequency Change after BRV Treatment in Switching Group	n = 85 (%)
- Seizure Freedom	26 (30.6)
- Seizure Frequency Responder ≥ 50%	41 (48.2)
- Seizure Frequency Responder < 50%	9 (10.6)
- Seizure Frequency Unchanged	8 (9.4)
- Seizure Frequency Aggravation	1 (1.2)

**Table 5:** Adverse events.

Adverse Events	Baseline, n = 156 (%)
- Yes	26 (16.6)
- No	130 (83.3)
**Types of Adverse Events**	**n = 156 (%)**
- Non-Behavioral Adverse Events	18 (11.5)
- Behavioral Adverse Events	8 (5.1)

**Table 6:** Adverse events in patients switching from LEV.

Switching Patients	n = 85 (%)
-No Adverse Events	75 (88.2)
-Behavioral Adverse Events	3 (3.5)
-Non-Behavioral Adverse Events	7 (8.2)

## Data Availability

Data were not reported.

## References

[1] Moshé S.L., Perucca E., Ryvlin P., Tomson T. (2015). Epilepsy: New advances. Lancet.

[2] Brodie M.J., Barry S.J.E., Bamagous G.A., Norrie J.D., Kwan P. (2012). Patterns of treatment response in newly diagnosed epilepsy. Neurology.

[3] Malek N., Heath C.A., Greene J. (2017). A review of medication adherence in people with epilepsy. Acta Neurol. Scand..

[4] Gillard M., Fuks B., Leclercq K., Matagne A. (2011). Binding characteristics of brivaracetam, a selective, high affinity SV2A ligand in rat, mouse and human brain: Relationship to anti-convulsant properties. Eur. J. Pharmacol..

[5] UCBPharma (2018). Briviact®(brivaracetam) Summary of Product Characteristics. https://www.ema.europa.eu/en/documents/product-information/briviact-epar-product-information_en.pdf.

[6] Biton V., Berkovic S.F., Abou-Khalil B., Sperling M.R., Johnson M.E., Lu S. (2014). Brivaracetam as adjunctive treatment for uncontrolled partial epilepsy in adults: A phase III randomized, double-blind, placebo-controlled trial. Epilepsia.

[7] Ryvlin P., Werhahn K.J., Blaszczyk B., Johnson M.E., Lu S. (2014). Adjunctive brivaracetam in adults with uncontrolled focal epilepsy: Results from a double-blind, randomized, placebo-controlled trial. Epilepsia.

[8] Klein P., Schiemann J., Sperling M.R., Whitesides J., Liang W., Stalvey T., Brandt C., Kwan P. (2015). A randomized, double-blind, placebo-controlled, multicenter, parallel-group study to evaluate the efficacy and safety of adjunctive brivaracetam in adult patients with uncontrolled partial-onset seizures. Epilepsia.

[9] Kwan P., Trinka E., Van Paesschen W., Rektor I., Johnson M.E., Lu S. (2014). Adjunctive brivaracetam for uncontrolled focal and generalized epilepsies: Results of a phase III, double-blind, randomized, placebo-controlled, flexible-dose trial. Epilepsia.

[10] Strzelczyk A., Klein K.M., Willems L.M., Rosenow F., Bauer S. (2016). Brivaracetam in the treatment of focal and idiopathic generalized epilepsies and of status epilepticus. Expert Rev. Clin. Pharmacol..

[11] Yates S.L., Fakhoury T., Liang W., Eckhardt K., Borghs S., D’Souza J. (2015). An open-label, prospective, exploratory study of patients with epilepsy switching from levetiracetam to brivaracetam. Epilepsy Behav..

[12] Fonseca E., Guzmán L., Quintana M., Abraira L., Santamarina E., Salas-Puig X., Toledo M. (2020). Efficacy, retention, and safety of brivaracetam in adult patients with genetic generalized epilepsy. Epilepsy Behav..

[13] Ben-Menachem E., Mameniškienė R., Quarato P.P., Klein P., Gamage J., Schiemann J., Johnson M.E., Whitesides J., McDonough B., Eckhardt K. (2016). Efficacy and safety of brivaracetam for partial-onset seizures in 3 pooled clinical studies. Neurology.

[14] Walker M.C., Sander J.W. (1997). Difficulties in extrapolating from clinical trial data to clinical practice: The case of antiepileptic drugs. Neurology.

[15] Steinhoff B.J., Staack A.M., Hillenbrand B.C. (2017). Randomized controlled antiepileptic drug trials miss almost all patients with ongoing seizures. Epilepsy Behav..

[16] Steinig I., von Podewils F., Möddel G., Bauer S., Klein K.M., Paule E., Reif P.S., Willems L.M., Zöllner J.P., Kunz R. (2017). Postmarketing experience with brivaracetam in the treatment of epilepsies: A multicenter cohort study from Germany. Epilepsia.

[17] Hirsch M., Hintz M., Specht A., Schulze-Bonhage A. (2018). Tolerability, efficacy and retention rate of brivaracetam in patients previously treated with levetiracetam: A monocenter retrospective outcome analysis. Seizure.

[18] Villanueva V., López-González F.J., Mauri J.A., Rodriguez-Uranga J., Olivé-Gadea M., Montoya J., Ruiz-Giménez J., Zurita J., The BRIVA-LIFE Study Group (2019). BRIVA-LIFE—A multicenter retrospective study of the long-term use of brivaracetam in clinical practice. Acta Neurol. Scand..

[19] Matagne A., Margineanu D.G., Kenda B., Michel P., Klitgaard H. (2008). Anti-convulsive and antiepileptic properties of brivaracetam (ucb 34714), a high-affinity ligand for the synaptic vesicle protein, SV2A. Br. J. Pharmacol..

[20] Adewusi J., Burness C., Ellawela S., Emsley H., Hughes R., Lawthom C., Maguire M., McLean B., Mohanraj R., Oto M. (2020). Brivaracetam efficacy and tolerability in clinical practice: A UK-based retrospective multicenter service evaluation. Epilepsy Behav..

[21] Gillis R.M., Wammes-van der Heijden E.A., Schelhaas H.J., Tan I.Y., Festen D.A., Majoie M.H. (2020). Efficacy and tolerability of brivaracetam in patients with intellectual disability and epilepsy. Acta Neurol. Belg..

[22] Matagne A., Kenda B., Michel P., Klitgaard H. (2003). UCB 34714, a new pyrrolidone derivative, suppresses seizures epileptogenesis in animal models of chronic epilepsy in vivo. Epilepsia.

[23] Trenité D.K.N., Genton P., Parain D., Masnou P., Steinhoff B.J., Jacobs T., Pigeolet E., Stockis A., Hirsch E. (2007). Evaluation of brivaracetam, a novel SV2A ligand, in the photosensitivity model. Neurology.

[24] Zhu L.N., Chen D., Chen T., Xu D., Chen S.H., Liu L. (2017). The adverse event profile of brivaracetam:a meta-analysis of randomized controlled trials. Seizure.

[25] Rigo J., Nguyen L., HANS G., Belachew S., Moonen G., Klitgaard H. (2004). UCB 34714: Effect on inhibitory and excitatory neurotransmission. Epilepsia.

[26] Carunchio I., Pieri M., Ciotti M.T., Albo F., Zona C. (2007). Modulation of AMPA receptors in cultured cortical neurons induced by the antiepileptic drug levetiracetam. Epilepsia.

[27] Kwan P., Arzimanoglou A., Berg A.T., Brodie M.J., Allen Hauser W., Mathern G., Moshé S.L., Perucca E., Wiebe S., French J. (2010). Definition of drug resistant epilepsy: Consensus proposal by the ad hoc task force of the ILAE Commission on Therapeutic Strategies. Epilepsia.

